# Melatonin Attenuates Intermittent Hypoxia-Induced Lipid Peroxidation and Local Inflammation in Rat Adrenal Medulla

**DOI:** 10.3390/ijms151018437

**Published:** 2014-10-13

**Authors:** Yu Liu, George Lim Tipoe, Man Lung Fung

**Affiliations:** 1 Department of Physiology, Li Ka Shing Faculty of Medicine, The University of Hong Kong, Hong Kong, SAR, China; E-Mail: hannahliu8383@gmail.com; 2 Faculty of Medicine, Shenzhen University, Shenzhen 518060, Guangdong, China; 3 Department of Anatomy, Li Ka Shing Faculty of Medicine, The University of Hong Kong, Hong Kong, SAR, China; E-Mail: tgeorge@hkucc.hku.hk

**Keywords:** chronic intermittent hypoxia, cardiovascular, cellular injury, anti-oxidant

## Abstract

Chronic intermittent hypoxia (CIH) induces lipid peroxidation and leads to cardiovascular dysfunction, in which impaired activities of the adrenal medulla are involved. This may be caused by CIH-induced injury in the adrenal medulla, for which the mechanism is currently undefined. We tested the hypothesis that melatonin ameliorates the CIH-induced lipid peroxidation, local inflammation and cellular injury in rat adrenal medulla. Adult Sprague–Dawley rats were exposed to air (normoxic control) or hypoxia mimicking a severe recurrent sleep apnoeic condition for 14 days. The injection of melatonin (10 mg/kg) or vehicle was given before the daily hypoxic treatment. We found that levels of malondialdehyde and nitrotyrosine were significantly increased in the vehicle-treated hypoxic group, when compared with the normoxic control or hypoxic group treated with melatonin. Also, the protein levels of antioxidant enzymes (superoxide dismutase (SOD)-1 and SOD-2) were significantly lowered in the hypoxic group treated with vehicle but not in the melatonin group. In addition, the level of macrophage infiltration and the expression of inflammatory cytokines (tumor necrosis factor (TNF)-α, interleukin (IL)-1β and IL-6) and mediators (inducible nitric oxide synthase (iNOS), cyclooxygenase-2 (COX-2)) were elevated in the vehicle-treated hypoxic group, but were significantly ameliorated by the melatonin treatment. Moreover, the amount of apoptotic cells in the hypoxic groups was significantly less in the melatonin-treated group. In conclusion, CIH-induced lipid peroxidation causes local inflammation and cellular injury in the adrenal medulla. The antioxidant and anti-inflammatory actions of melatonin are indicative of a protective agent against adrenal damage in patients with severe obstructive sleep apnea syndrome.

## 1. Introduction

Patients with sleep-disordered breathing suffer from recurrent episodes of apnea/hypopnea caused by obstruction of the upper airway and/or lowered central ventilatory activities. It is known that patients with obstructive sleep apnea (OSA) syndrome have increased risks of developing hypertension, cardiovascular diseases and neuro-cognitive deficits [1,2,3]. Studies have also shown that recurrent episodes of apnea/hypopnea cause chronic intermittent hypoxia (CIH), which induces lipid peroxidation and subsequently causes the pathophysiological change in the OSA patient [4,5,6]. Indeed, we have shown that CIH-induced lipid peroxidation plays a significant pathogenic role in the hippocampal injury in a rat model with chronic exposure to intermittent hypoxia mimicking a severe OSA condition, which underpins the involvement of overproduction of reactive oxygen species (ROS) and free radicals in apoptotic cell death and neuronal dysfunction [7,8]. In addition, it has been shown that that cellular injuries caused by lipid peroxidation lead to inflammation, which induces the inflammatory response that could be an important contributing factor in promoting cardiovascular morbidity in OSA patients [9,10]. In fact, lipid peroxidation and ROS are potent inducers of inflammatory pathways that promote the expression of adhesion molecules and pro-inflammatory cytokines [11]. A growing amount of evidence supports pathophysiological roles of CIH-induced lipid peroxidation and inflammation in cardiovascular morbidities in OSA patients [12,13,14].

The adrenal medulla is an important effector for the autonomic regulation of the cardiovascular response to hypoxia [15,16]. The production and release of catecholamines from the adrenomedullary chromaffin cells are not only crucial to the cardiovascular response to hypoxia [17,18] but also to the proper respiratory and metabolic responses to asphyxia [19]. In OSA patients, elevated levels of the sympathetic activity and circulating catecholamines are indicative of pathophysiological changes in the adrenal medulla. However, there is paucity information on the lipid peroxidation and cellular injuries in the adrenal medulla, if any, and its pathogenic mechanism under CIH conditions.

Melatonin, which is secreted from the pineal gland, possesses antioxidant and anti-inflammatory properties at both physiological and pharmacological concentrations [20,21,22,23,24]. Pharmacological doses of exogenously administered melatonin are known to be protective against free radicals [25] shown in a number of pathological conditions such as hypoxia-induced hepatic injury and the ischemic-reperfusion injuries in the skeletal muscle, heart, and brain [26,27,28,29]. In this study, we aimed to examine the hypothesis that melatonin is protective against the lipid peroxidation, local inflammation and cellular injury induced by CIH in the adrenal medulla in rats.

## 2. Results

**Figure 1:**
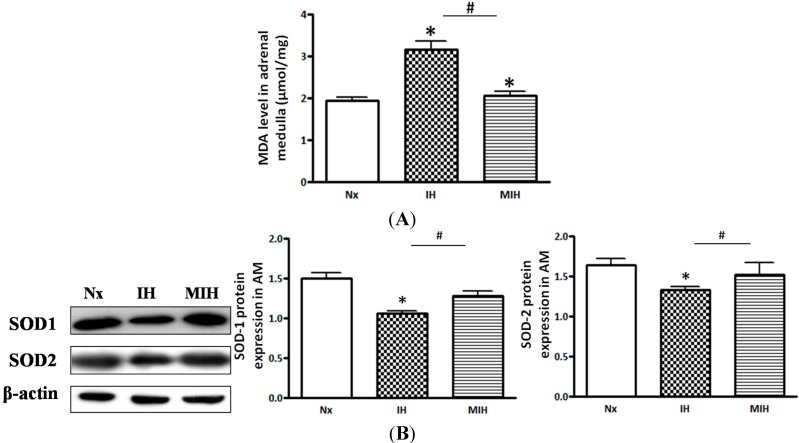
Levels of malondiadehyde (MDA) and the protein expression of antioxidant enzymes in adrenal medulla of hypoxic rats and controls. (**A**) The level of MDA was significantly increased in the IH group when compared with the normoxic control and hypoxic group co-treated with melatonin; and (**B**) antioxidant enzymes protein expression was significantly lower in the IH group than those of the normoxic control and MIH groups (* *p* < 0.05, *versus* normoxic control; # *p* < 0.05, *versus* IH group. *n* = 6 per group). Nx, normoxic control; IH, intermittent hypoxia; MIH, melatonin-treated intermittent hypoxia.

The malondialdehyde (MDA) level in the adrenal medulla was significantly increased in the hypoxic group treated with vehicle, when compared with that of the normoxic control. The MDA level was remarkably decreased to the normoxic level by the melatonin treatment (Figure 1A). The protein expressions of the antioxidant enzymes, superoxide dismutase (SOD)-1 and SOD-2 in the adrenal medulla were examined by Western blot study. The protein levels of SOD1 and SOD2 were significantly lowered in the vehicle-treated hypoxic group (Figure 1B).

**Figure 2:**
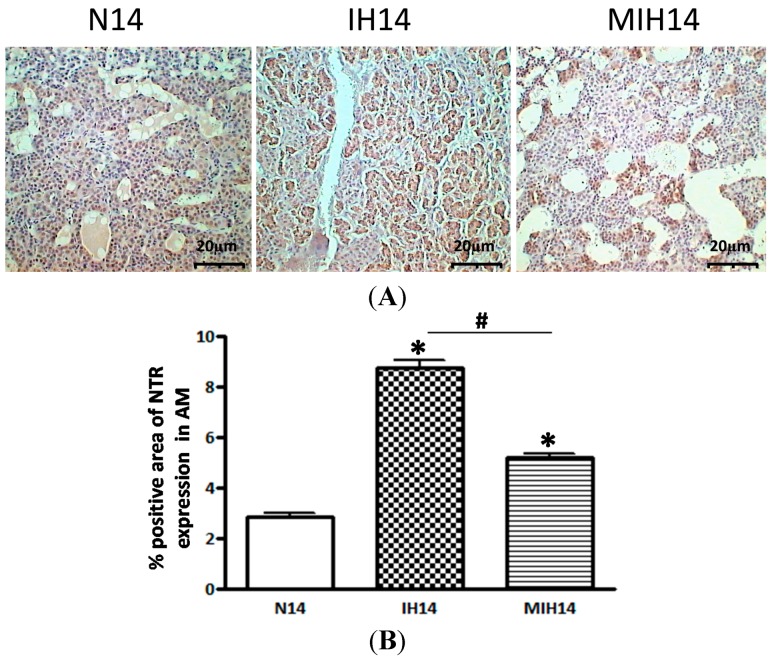
Immunostaining of nitrotyrosine (NTR) in the adrenal medulla of hypoxic rats and controls. (**A**) The positive-immunoreactivity of NTR was significantly increased in the hypoxic groups (IH and MIH group) than the Nx, but it was attenuated by the melatonin treatment (MIH group); and (**B**) Data are presented in % area with positive staining of NTR immunoreactivity. (* *p* < 0.05, *versus* normoxic control; # *p* < 0.05, *versus* IH group. *n* = 6 per group). Nx, normoxic control; IH, intermittent hypoxia; MIH, melatonin-treated intermittent hypoxia.

Immunohistochemical studies revealed that the immunoreactivity of nitrotyrosine (NTR) was present in the adrenal medulla. Image analysis measuring the % adrenal medulla area with positive-immunostaining of NTR showed a significant increase in the NTR level in the hypoxic groups when compared with the normoxic control. The NTR level in melatonin-treated hypoxic group was significantly lower than that of the vehicle-treated group (Figure 2). These results suggest an increased level of reactive nitrogen species (RNS) caused by CIH-induced oxidative stress in the adrenal medulla under CIH condition, which could be mitigated by melatonin treatment.

**Figure 3:**
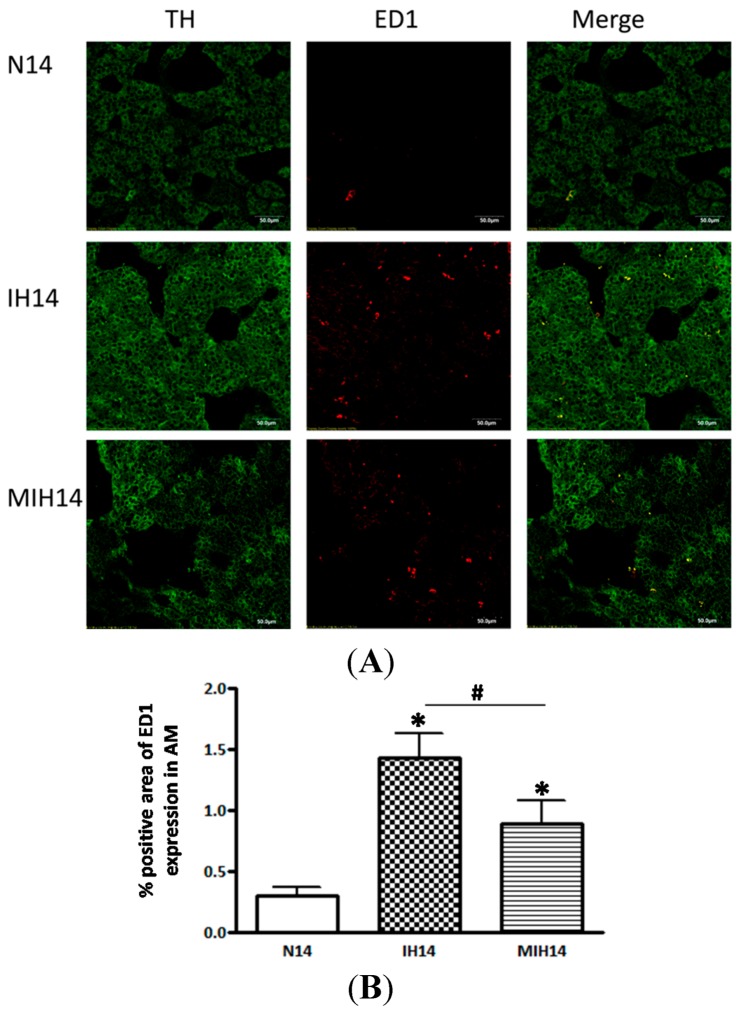
The localization and expression of ED1-containing macrophages and tyrosine hydroxylase (TH) in the adrenal medulla of hypoxic rats and controls. (**A**) The ED1 (red) and TH (green) expression was significantly more in the hypoxic groups (IH and MIH group) than the Nx, but it was attenuated by the melatonin treatment (MIH group); and (**B**) Data are presented in % area with positive staining of ED1 immunoreactivity. (* *p* < 0.05, *versus* normoxic control; # *p* < 0.05, *versus* IH group. *n* = 6 per group). Nx, normoxic control; IH, intermittent hypoxia; MIH, melatonin-treated intermittent hypoxia.

To examine the infiltration of macrophages in the adrenal medulla, double-staining of cellular markers for macrophages, anti-CD68 antibody (ED1), and for adrenochromaffin cells, and tyrosine hydroxylase (TH), was performed. Results showed that the sectional area with ED1-positive staining (%) was significantly increased in the hypoxic groups but the increase was much less in the melatonin-treated hypoxic group (Figure 3), indicating an increase in the infiltration of macrophages in the adrenal medulla under CIH condition, which could be attenuated by the melatonin treatment.

**Figure 4:**
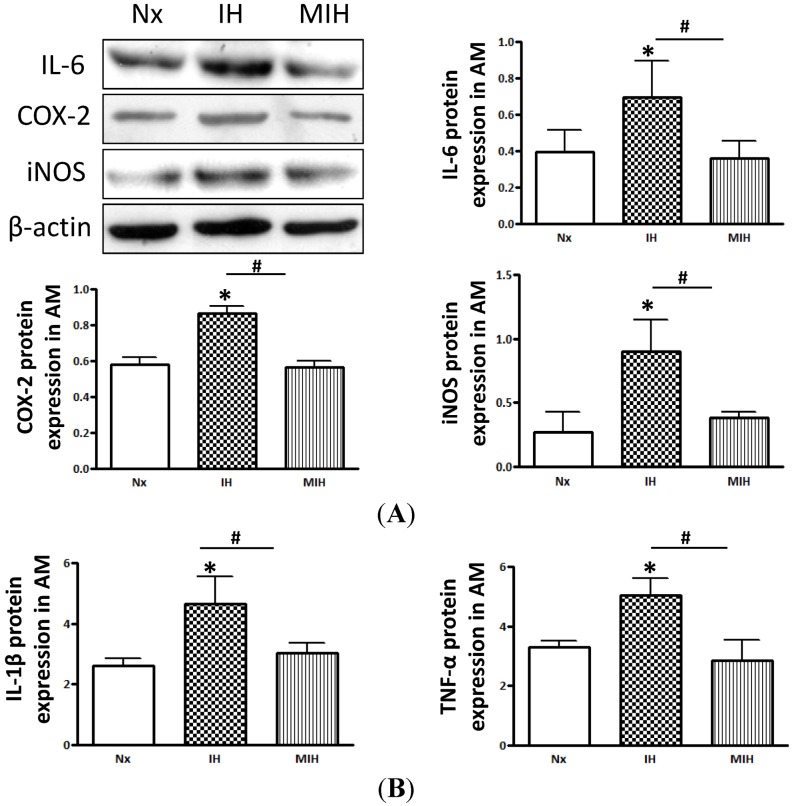
Levels of the protein expression of pro-inflammatory cytokines and mediators in adrenal medulla. (**A**) Protein expression of pro-inflammatory mediators were significantly higher in the IH group than those of the normoxic control and MIH groups; and (**B**) Protein expression of pro-inflammatory cytokines characterized by ELISA. (* *p* < 0.05, *versus* normoxic control; # *p* < 0.05, *versus* IH group. *n* = 6 per group). Nx, normoxic control; IH, intermittent hypoxia; MIH, melatonin-treated intermittent hypoxia.

The protein expressions of inflammatory cytokines, namely interleukin (IL)-1β, IL-6 and tumor necrosis factor (TNF)-α, and mediators including cyclooxygenase-2 (COX-2) and inducible nitric oxide synthase (iNOS), in the adrenal medulla were examined by Western blotting study and ELISA assay. The expression levels of the inflammatory cytokines (IL-1β, IL-6 and TNF-α) and mediators (iNOS and COX-2) were significantly higher in the vehicle-treated hypoxic group than those in the normoxic and melatonin-treated group (Figure 4).

**Figure 5:**
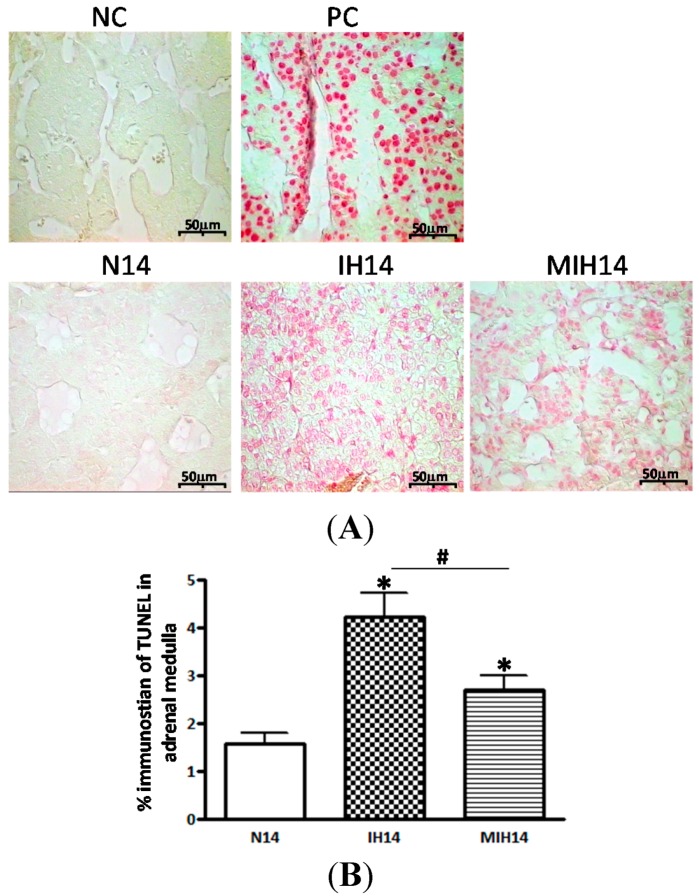
TUNEL-staining of apoptotic cells in the adrenal medulla was increased in the hypoxic groups (IH and MIH group) but the increase was less in the MIH group. (**A**) TUNEL-staining in sections of the adrenal medulla in Nx, IH and MIH groups;and (**B**) Summary of % TUNEL-staining in the adrenal medulla. Calibration bar is 20 μm. (* *p* < 0.05, *versus* normoxic control; # *p* < 0.05, *versus* IH group. *n* = 6 per group). NC, negtive control; PC, positive control; Nx, normoxic control; IH, intermittent hypoxia; MIH, melatonin-treated intermittent hypoxia.

The amount of apoptotic cells in the adrenal medulla was assessed by TUNEL assay. The sectional area with TUNEL-positive staining (%) was significantly increased in the hypoxic groups but the increase was much less in the melatonin-treated hypoxic group (Figure 5), suggesting that melatonin is protective against CIH-induced cell death in the adrenal medulla.

## 3. Discussion

**Figure 6:**
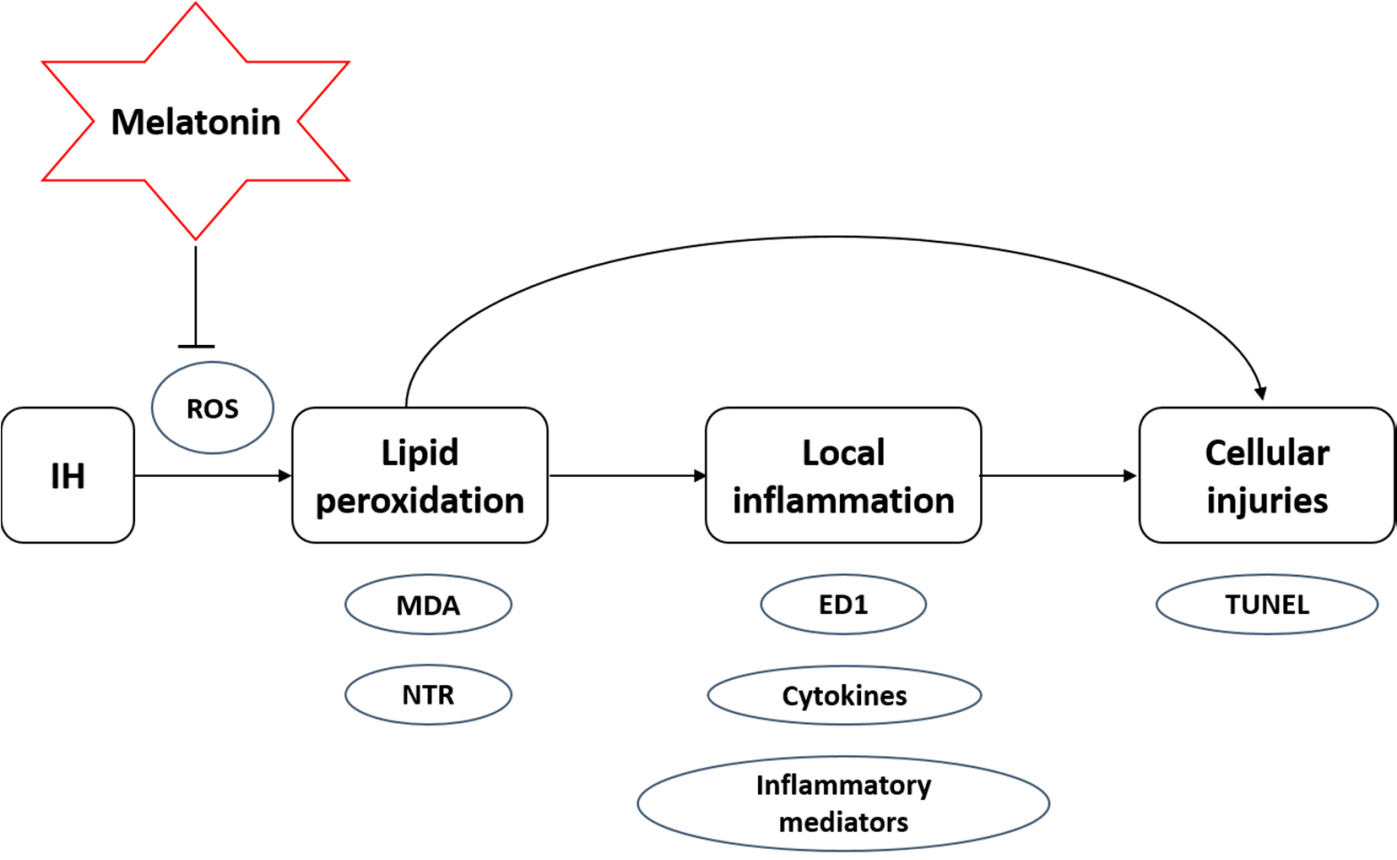
Melatonin ameliorates the cellular injuries in the adrenal medulla mediated by lipid peroxidation and inflammation induced by intermittent hypoxia.

In this study, we demonstrated that the CIH exposure mimicking a severe OSA condition in rats induces lipid peroxidation, local inflammation and apoptotic cell death in the adrenal medulla with decreased expressions of antioxidant enzymes (Figure 6). We also showed that the administration of melatonin significantly ameliorated the CIH-induced lipid peroxidation and local inflammation. Lipid peroxidation is one of the major processes mediating the breakdown of membrane lipids leading to cellular injuries caused by free radicals during oxidative insults [30,31]. The MDA level is elevated when the membrane lipoproteins and polyunsaturated fatty acids are attacked by free radicals, which estimates the level of lipid peroxidation [32,33,34]. Although it is not a direct measurement of oxidative stress, we found an increased MDA level in the adrenal medulla of hypoxic rats, suggesting elevated levels of lipid peroxidation, which could be mediated by CIH-induced oxidative stress. This is consistent with our previous studies reporting antioxidant melatonin attenuated the elevated MDA levels and cellular injuries induced by CIH-induced lipid peroxidation [7,8]. Also, our results showed the increased level of NTR-immunoreactivity in the adrenal medulla of CIH rats. Since the NTR is an indicator of cellular injuries and inflammation mediated by RNS and ROS in oxidative stress [35,36], the increased NTR level suggests that the CIH-induced oxidative stress could lead to the injury of adrenochromaffin cells in the adrenal medulla. This is supported by the finding that apoptotic cells were significantly more in the adrenal medulla in the hypoxic groups. Furthermore, antioxidant enzymes are important cellular defenders against oxidative stress [37]. The finding of a significant decrease in the protein expression of antioxidant enzymes in the adrenal medulla of hypoxic rats indicates an involvement of lowered antioxidant capacity in the CIH-induced lipid peroxidation, leading to the local inflammation and cell death in the adrenal medulla.

Inflammation plays an important role in the pathogenesis of tissue injury in response to lipid peroxidation in the adrenal medulla. We found an increase in the infiltration of macrophage in the adrenal medulla of CIH rats, which was attenuated by melatonin treatment. This suggests a local inflammation in the adrenal gland with activated macrophages, which are known to be capable of producing pro-inflammatory cytokines, free radicals and myeloperoxidase in inflamed tissues. In addition, macrophages could play an important role in the removal of necrotic debris and apoptotic cells. Also, activated macrophages are responsible for the release of growth factors for angiogenesis and turnover of extra-cellular matrix, which are vital requisites for the tissue healing and regeneration. Furthermore, the induction of the expressions of inflammatory mediators, including COX-2 and iNOS, and cytokines IL-1β, IL-6, and TNF-α, play important roles in the tissue and cellular injuries under lipid peroxidation [38,39,40,41]. In fact, the protein expressions of these inflammatory mediators were consistently increased in the vehicle-treated hypoxic group, suggesting that there is a close link between lipid peroxidation and inflammation during hypoxia. These could cause apoptotic cell death mediated by a mitochondria-mediated intrinsic apoptotic pathway, characterized by the activation of caspases induced by lipid peroxidation under CIH conditions [42]. Thus, oxidative insults mediated by locally produced ROS, and mitochondrial release of cytochrome c could activate caspase-3, leading to apoptosis in the adrenal medulla [43].

Composed mainly of hormone-producing chromaffin cells, the adrenal medulla is the principal site of the conversion of the amino acid tyrosine into the catecholamines epinephrine, norepinephrine, and dopamine. Studies in both humans and experimental models have shown that CIH leads to hypertension as well as increased circulating catecholamines [44,45,46]. Recently, studies have shown that CIH increases the release of catecholamines from adrenal chromaffin cells via ROS-mediated activation of protein kinase C (PKC) and enhanced neuropeptide Y synthesis by ROS, thus elevating the circulating catecholamines and arterial pressures [15,18,47]. Also, Peng *et al.* [48] reported that the muscarinic acetylcholine receptor-mediated calcium influx in the adrenal medulla plays a role in the oxidative stress and HIF-α isoform imbalance mediated by CIH-induced sympathetic activation, resulting in the activation of mammalian target of rapamycin pathway and calpain proteases. In this context, the increased level of lipid peroxidation and inflammation may also be involved in the pathological cascade leading to elevated release of catecholamines in the adrenal medulla under CIH conditions.

Melatonin is a potent ROS scavenger which exerts antioxidant effects against CIH-induced lipid peroxidation. Indeed, our results showed that administration of melatonin significantly decreased the amount of MDA and NTR and also markedly reduced the expressions of these inflammatory cytokines and mediators in the adrenal medulla of the hypoxic rat. This is in consistent with our previous studies showing the protective effect of melatonin against CIH-induced neuronal and cardiac injuries [7,8]. The known neuroprotective effect of melatonin is, in part, attributed to its antioxidant property against the lipid peroxidation in the central nervous system during oxidative insults [49,50,51]. In fact, melatonin, which directly scavenges ROS, also reduces lipid peroxidation indirectly through mediating an up-regulation of the expression of antioxidant enzymes [52,53]. In addition, recent evidence showed that melatonin modulates neuro-inflammation by inhibiting the NF-κB pathway and downstream mediators of inflammation. Also melatonin protects against lipid peroxidation and inflammation by upregulating the nuclear erythroid 2-related factor 2 (Nrf2) pathway [54]. Besides, melatonin can inhibit the apoptosis by acting on the abundance of the two main members of the Bcl-2 family [55,56,57,58,59,60]. Thus, the potent antioxidant and anti-inflammatory properties of melatonin are central to its protective effects against cellular injuries in the adrenal medulla.

In conclusion, our results support the hypothesis that CIH-induced lipid peroxidation is involved in local inflammation and apoptosis in the rat adrenal medulla. Administration of melatonin, as an antioxidant agent, could prevent the CIH-induced tissue injury and local inflammation in the adrenal medulla.

## 4. Material and Methods

### 4.1. Treatment of Chronic Intermittent Hypoxia in Rats

Healthy Sprague–Dawley rats age 28 days (~150 g weight) were randomly divided into two groups, namely normoxic control (Nx), and CIH (*n* = 6). While the Nx rats were maintained in room air, CIH rats were kept in acrylic chambers for normobaric hypoxia in the same room and had free access to water and regular chow. The oxygen fraction inside the chamber was cyclic between 5% ± 0.5% and 21% ± 0.5% per min, 8 h per day diurnally. The desired oxygen content was established by a mixture of room air and nitrogen that was regulated and monitored by an oxygen analyzer (Vacumetrics Inc., Ventura, CA, USA). The rats were exposed to CIH for 14 days and then were immediately sacrificed after being removed from the chambers.

### 4.2. Drug Preparation

Melatonin (Sigma, St. Louis, MO, USA) solution was prepared freshly before injection by dissolving the indoleamine in absolute ethanol and further dilution with normal saline; the final concentration of ethanol was 2%. Melatonin in 10 mg/kg body weight or vehicle (2% ethanol in normal saline) was administered intraperitoneally each day 30 min before hypoxic exposure.

### 4.3. Measurement of Malondialdehyde (MDA) Formation

The MDA level was determined using a BIOXYTECH LPO-586™^®^ kit (OxisResearch, Scottsdale, OR, USA). The reaction product was measured spectrophotometrically at 586 nm. Standard curves were constructed with 1,1,3,3-tetraethoxypropane as a standard. The MDA concentration (μM) in adrenal medulla was normalized to wet tissue weight (mg) and expressed as μmol/mg.

### 4.4. Immunohistochemistry

Following deep anesthesia with halothane, the rat was decapitated and the adrenal gland was excised rapidly. The adrenal medulla was carefully dissected out and was fixed in neutral buffered formalin for 72 h. Tissues were processed routinely for histology and embedded in paraffin blocks. Serial sections of 5 µm thickness were cut and mounted on silanized slides (Dako Denmark A/S, Denmark). Sections were kept in the oven overnight at 56 °C. Consequently, sections were dewaxed with xylene and rehydrated with a series of decreasing grades of ethanol solution. Sections were immunostained with antiserum to nitrotyrosine (NTR) (goat polyclonal antibody, 1:500 dilution, Santa Cruz Biotechnology, Santa Cruz, CA, USA), using LSAB kit (K0690, Dako Denmark A/S, Dako, Denmark) and were deparaffinized and rehydrated. Sections were immersed in antigen retrieval solution (0.1 M citric acid buffer, pH 6.0) for 10 min at 98 °C. To block endogenous peroxidase activity, the sections were immersed in 3% hydrogen peroxide for 5 min at room temperature. Sections were pre-incubated with 20% normal serum for 2 h to reduce non-specific binding for the anti-serum. Then sections were incubated with the corresponding primary antibodies in 0.05 M Tris–HCl buffer, respectively, containing 2% bovine serum albumin overnight at 4 °C. Followed by three times washing in PBS, the sections were incubated with biotinylated link agent and streptavidin peroxidase for 30 min at room temperature. Finally, sections were washed and the peroxidase was visualized by immersing in 0.05% diaminobenidine (DAB, Dako Denmark A/S, Dako, Denmark) containing 0.03% hydrogen peroxide in Tris–HCl buffer (pH 7.5) for 3–5 min. Mild counterstaining with hematoxylin was then performed. Positive staining was indicated by a brown color. Control sections were incubated with either normal mouse or rabbit IgG and stained uniformly negative.

### 4.5. Immunofluorescence Double Staining

Paraffin sections were processed for indirect immunofluorescent double staining as reported previously. Briefly, sections were incubated overnight at 4 °C with rabbit anti-rat ED1 serum, diluted to 1:80, followed with anti-tyrosine hydroxylase serum (Chemicon International Inc., Temecula, CA, USA), diluted to 1:100 overnight at 4 °C. After rinses in PBS, the primary antibodies were detected using anti-rabbit serum labeled with rhodamine (1:100, Jackson ImmunoResearch Laboratories Inc., West Grove, PA, USA) for ED1 and anti-sheep serum labeled with FITC (1:100, Jackson ImmunoResearch Laboratories Inc.) for TH at room temperature for 2 h. Positive immunoreactivity for ED1 (red) and for TH (green) was examined with a fluorescent microscope with a DC 200 digital camera (Leica Microsystems Ltd., Heerbrugg, Switzerland). For the control, primary antibodies were substituted with buffer and sections were incubated with rabbit non-immune serum.

### 4.6. Western Blotting

The membrane and cytosolic protein extractions were performed by using the ProteoExtract native membrane protein extraction kit (Merck KGaA, Darmstadt, Germany) and protocols of immunoblotting of proteins was described previously [8,61,62,63]. In brief, the SD rats were deeply anesthetized and decapitated. Adrenal glands were quickly removed and chilled in the ice-old PBS solution. The adrenal medulla were dissected and were immediately frozen in liquid nitrogen and stored at −70 °C. Samples were homogenized in the ice-cold extraction buffer I containing 10 μL protease inhibitor cocktail (Merck) and centrifuged at 16,000× *g* for 15 min at 4 °C. Supernatant enriched in cytosolic proteins was transferred to the sample tube. Pellet was added with ice-cold extraction buffer II containing 10 μL protease inhibitor cocktail. The pellet was resuspended gently using a pipette and incubated for 30 min at 4 °C. The samples were centrifuged at 16,000× *g* for 15 min at 4 °C. Supernatant containing membrane fraction and membrane protein was transferred completely into sample tubes. The amount of protein was determined using protein assay (Bio-Rad Laboratories, Hercules, CA, USA). Proteins were mixed with Laemmi buffer containing lysis buffer, 10% 2-mercaptoethanol, and 2 mg/mL bromophenol blue. Samples were incubated at 70 °C for 30 min and 60 μL of each sample was loaded in each well of a 7.5% SDS-polyacrylamide mini-gel. Membranes were then transferred to polyvinylidene difluoride membranes using a transblotting apparatus (Bio-Rad Laboratories) for 30 min. Then membranes were incubated at room temperature for 2 h in TBS buffer with 5% skimmed milk, followed by incubating with primary monoclonal antibody COX-2 (rabbit polyclonal antibody, 1:500 dilution, Cat # sc-7951, Santa Cruz), IL-6 (goat polyclonal antibody, 1:1000 dilution, Cat # sc-1265, Santa Cruz), iNOS (mouse monoclonal antibody, 1:500 dilution, Cat # sc-7271, Santa Cruz), SOD1 (rabbit polyclonal antibody, 1:1000 dilution, Cat # sc-11407, Santa Cruz) and SOD2 (rabbit polyclonal antibody, 1:1000 dilution, Cat # sc-30080, Santa Cruz) in TBS buffer with 5% skimmed milk for overnight at 4 °C. After incubation, membranes were washed and incubated with second antibody, anti-rabbit IgG conjugated to HRP for COX-2 and SOD1 (1:10,000), anti-goat IgG conjugated to HRP for SOD2 and IL-6 (1:10,000), anti-mouse IgG conjugated to HRP for iNOS (1:10,000) in TBS solution with 5% skimmed milk for 1 h. Then blots were developed using chemiluminescence reagent. Films were exposed and analyzed by using Image J software (National Institutes of Health, Bethesda, MD, USA). Results were expressed in relative optical density.

### 4.7. Enzyme-Linked Immunosorbent Assay (ELISA) Measurement

ELISA measurements of TNF-α and IL-1β were performed by using corresponding ELISA development kits from PeproTech (PeproTech Inc., Rocky Hill, NJ, USA) according to user instructions.

### 4.8. Apoptosis Measurement

To demonstrate the apoptotic cell death in the adrenal medulla, the terminal deoxynucleotidyl transferase-mediated dUTP-nick end labeling (TUNEL) assay, which detects 3' hydroxyl ends in fragmented DNA as an early event in apoptotic cascade, was used. After dewaxation and rehydration of the paraffin-embedded tissue sections, stainings were performed according to the manufacturer’s instructions regarding the TUNEL assay by using the *in situ* cell death detection kit (Roche Applied Science, Mannheim, Germany). DNase I recombinant in the sections was used as the positive control. Label solution without terminal transferase was used in place of TUNEL reaction mixture for negative control. The positive immunostainings of TUNEL were examined by light microscope (Zeiss Axiolab, Carl Zeiss Inc., Oberkochen, Germany).

### 4.9. Statistical Analysis

Results are presented as means ± S.E.M. and statistical analyses between groups are one-way ANOVA with post-hoc tests for multiple comparisons. Statistical significance was considered at *p* < 0.05.
